# Melatonin’s Impact on Antioxidative and Anti-Inflammatory Reprogramming in Homeostasis and Disease

**DOI:** 10.3390/biom10091211

**Published:** 2020-08-20

**Authors:** Diana Maria Chitimus, Mihaela Roxana Popescu, Suzana Elena Voiculescu, Anca Maria Panaitescu, Bogdan Pavel, Leon Zagrean, Ana-Maria Zagrean

**Affiliations:** 1 Division of Physiology and Neuroscience, Department of Functional Sciences, “Carol Davila” University of Medicine and Pharmacy, 010164 Bucharest, Romania; diana.chitimus@rez.umfcd.ro (D.M.C.); suzana.voiculescu@umfcd.ro (S.E.V.); bogdan.pavel@umfcd.ro (B.P.); leon.zagrean@umfcd.ro (L.Z.); 2 Department of Cardiology, “Carol Davila” University of Medicine and Pharmacy, Elias University Hospital, 010164 Bucharest, Romania; roxana.popescu@umfcd.ro; 3 Department of Obstetrics and Gynecology, “Carol Davila” University of Medicine and Pharmacy, Filantropia Clinical Hospital, 010164 Bucharest, Romania; anca.panaitescu@umfcd.ro

**Keywords:** melatonin, antioxidant, homeostasis, allostasis, maternal-fetal signaling, COVID 19, cardiovascular, neurodegenerative

## Abstract

There is a growing consensus that the antioxidant and anti-inflammatory properties of melatonin are of great importance in preserving the body functions and homeostasis, with great impact in the peripartum period and adult life. Melatonin promotes adaptation through allostasis and stands out as an endogenous, dietary, and therapeutic molecule with important health benefits. The anti-inflammatory and antioxidant effects of melatonin are intertwined and are exerted throughout pregnancy and later during development and aging. Melatonin supplementation during pregnancy can reduce ischemia-induced oxidative damage in the fetal brain, increase offspring survival in inflammatory states, and reduce blood pressure in the adult offspring. In adulthood, disturbances in melatonin production negatively impact the progression of cardiovascular risk factors and promote cardiovascular and neurodegenerative diseases. The most studied cardiovascular effects of melatonin are linked to hypertension and myocardial ischemia/reperfusion injury, while the most promising ones are linked to regaining control of metabolic syndrome components. In addition, there might be an emerging role for melatonin as an adjuvant in treating coronavirus disease 2019 (COVID 19). The present review summarizes and comments on important data regarding the roles exerted by melatonin in homeostasis and oxidative stress and inflammation related pathologies.

## 1. Introduction

Melatonin is a pineal hormone produced and released in relation to the circadian rhythm, while also synthesized in extrapineal tissues, like heart, liver, placenta, skin, kidney, gut, etc. [1,2,3,4]. Melatonin is an important regulator of physiologic processes and a guardian of body homeostatic balance. Its level varies during the day from 5 to 200 pg/mL [5].

Melatonin has *antioxidant* effects exerted through direct and indirect mechanisms that make this unrivaled multitasking molecule an endogenous protector against highly toxic oxygen- and nitrogen-derived free radicals. The main mechanisms of action attributed to melatonin are free radical scavenging, endogenous antioxidative enzymes stimulation, and improving the efficiency of other antioxidants. Melatonin’s particularity is that together with its metabolites, which act as antioxidants themselves, creates an antioxidant cascade that yields radical scavenger products [6], limiting the oxidative damage through a variety of mechanisms [7,8]. Thus, the radical quencher property of melatonin against the hydroxyl radical (OH) is superior to that of glutathione, while its action against the peroxyl radical (ROO) involves single electron transfer, hydrogen atom transfer, or radical adduct formation [8,9]. Besides lowering the amount of free radicals, melatonin can also interact with non-radical oxidants such as hydrogen peroxide (H_2_O_2_), singlet oxygen (^1^O_2_), and peroxynitrite (HNOO) [6,10]. Melatonin is effective in inhibiting metal-induced oxidation, as it was reported for copper, a redox generating metal, but it also chelates iron, lead, zinc, and aluminum [8]. 

**Figure 1:**
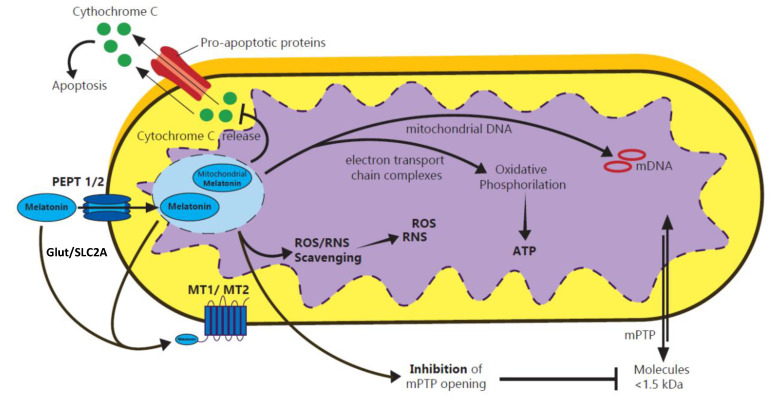
The roles of melatonin within the mitochondria. Melatonin is transported into mitochondria through PEPT1/2 oligopeptide and Glut/SLC2A transporters, but it is also synthesized within mitochondria [12,13]. Melatonin lowers the formation of free radicals and protects ATP synthesis at the mitochondrial level. It scavenges free oxygen (ROS) and nitrogen (RNS) reactive species, by preventing mitochondrial apoptosis and disruption of the electron transport chain. Melatonin interacts with MT1 and MT2 melatonin receptors, inhibits pro-apoptosis protein synthesis, and the subsequently cytochrome C leakage at the level of the membrane. It also protects mitochondrial DNA and prevents the opening of the mitochondrial permeability transition pore (mPTP) [13].

Along with its free radical scavenging properties, melatonin protects the mitochondria against oxidative stress by influencing the mitochondrial membrane potential, thus facilitating electron transfer antioxidant processes within the cell [11]. The roles of melatonin within mitochondria are exemplified in Figure 1.

Melatonin works through both receptor-independent and receptor-dependent antioxidant processes. Through its receptor-mediated actions, melatonin either inhibits pro-oxidative enzymes such as xanthine oxidase or enhances the activity of superoxide dismutase (SOD), glutathione peroxidase, and catalase [12,13]. 

Growing evidence supports the *anti-inflammatory* role of melatonin, both in acute and chronic inflammation processes. However, most of the data are obtained from in vitro and in vivo experimental studies, while clinical studies have proven inconsistent results [14]. Administration of exogenous melatonin in animal studies, prior to acute conditions, have shown a decrease in the inflammatory response, a reduction of pro-inflammatory cytokines, interleukine-1β (IL-1β), and tumor necrosis factor-α (TNF-α), and an increase in anti-inflammatory cytokine IL-4 levels in serum [15,16]. In addition, melatonin inhibits the expression of cyclooxygenase (COX) and inducible nitric oxide synthase (iNOS), while reducing the production of high concentrations of prostanoids and leukotrienes, as well as other mediators of the inflammatory process, such as chemokines and adhesion molecules [17,18].

Melatonin also exerts an *anti-apoptotic* effect that depends on its capacity to optimize the mitochondrial function, through antioxidative mechanisms. Some of the anti-apoptotic effects of melatonin are associated with an increase in the anti-apoptotic factor Bcl2, and a decrease in the pro-apoptotic factors Bax and caspase 3 [19,20,21]. However, an anti-angiogenic and *pro-apoptotic* effect through vascular endothelial growth factor (VEGF), hypoxia-induced factor 1α (HIF1α), Janus kinase 2/signal transducers and activators of transcription 3 (JAK/STAT3) inhibition, was observed in hepatic carcinoma [22]. It seems that melatonin tips the scales of the pro/anti-apoptotic balance according to local needs, in order to maintain homeostasis.

**Figure 2:**
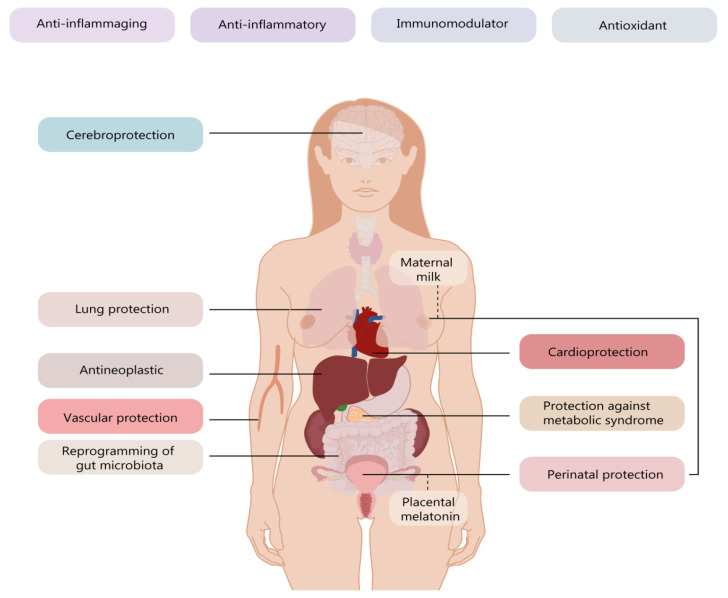
The widespread effects of melatonin at organ and system level.

Melatonin has been shown to have *pleiotropic* effects in numerous neurology, endocrinology, cardiology, fetal medicine, and oncology studies [20,23]. Moreover, its protective and allostatic effect spreads over many organs and systems (Figure 2).

The allostatic load implies the incapacity of the body systems to effectively adapt and cooperate in order to discard the effect of external stressors. Chronodisruption and sleep-deprivation elevate the allostatic load by altering the biological rhythms of hormones.

Melatonin plays an important role in establishing continuous chrono-biological homeostasis, as revealed by the increased load of allostasis in circadian disruptive patterns. Hunter et al. described a 33.8% reduction in melatonin production in night-shift workers over 24 h. Although there is no clear connection between increased cancer incidence, overall mortality, chronodisruption, and melatonin suppression, multiple studies reported a significant increment in breast or ovarian cancer in night-workers. Moreover, shift workers display a slow adaptation over the following week and have a lower night-time melatonin blood concentration [24]. This might explain why insufficient melatonin production favors an overload of stress and/or inefficient management of the body resources.

As melatonin has a rather short half-life (0.57–0.67 h), the need for longer acting substances has prompted the synthesis of analogues such as ramelteon, piromelatine, agomelatine, and tasimelteon. Ramelteon has a half-life of 1.5–2 h, with a metabolite with an even longer half-life (2–5 h) [25]. Ramelteon has a higher affinity for melatonin receptors (3–16 fold than melatonin) and is recommended in 8 mg dose for onset insomnia, decreasing sleep latency, and increasing sleep duration. Agomelatine and tasimelteon reach peak levels after 1–2 h and 0.5–3 h, respectively [25]. Agomelatine is indicated in treating sleep disorders associated to major depressive disorder. Its dose is 25 mg and has good oral absorption (80%). Tasimelteon is indicated in non-24-h sleep-wake disorder, in a dose of 20 mg [26,27].

With regard to exogenous melatonin administration and its side effects, up to date, only few studies focused on investigating possible adverse reactions as their primary objectives. Andersen et al. summarized in their analysis the safety of melatonin administration in humans, in both infants and adults. The most common side effects reported throughout literature were dizziness, headache, nausea, and sleepiness, while the long-term administration registered few to none adverse reactions [28].

In addition to the previously mentioned attributes, melatonin is intimately linked to reproduction and its most prominent role is to mediate reproductive effects in mammals that breed seasonally. In humans, melatonin influences reproduction as suggested by the widespread distribution of its receptors in reproductive organs and systems. Melatonin modulates the production and function of the human gonadotropins and steroid hormones and is considered to influence the onset of puberty, sexual maturation, follicular genesis and ovulation, pregnancy, and menopause [29]. In late pregnancy, melatonin concentration increases steadily towards the last trimester, returning to normal values after delivery [30,31]. The embryo and the fetus express receptors for melatonin since early stages of pregnancy suggesting that melatonin plays a crucial role in normal development during intrauterine life.

In the early stages of embryo development, the DNA suffers dynamic changes through active and passive demethylation and de novo methylation [32,33], crucial for fertilization, development, and tissue differentiation [34]. The rate-limiting enzyme for melatonin production, aralkylamine *N*-acetyltransferase *(Aanat*), mostly identified at the mitochondrial level, was linked to DNA demethylation. Thus, *Aanat* knockdown inhibited ten-eleven-translocation (TET) methylcytosine dioxygenase 2 (TET2) expression and DNA demethylation in the blastocyst, severely altering embryonic development and cellular differentiation [35]. These effects were counteracted by melatonin supplementation [36]. 

Melatonin was also explored as a neuroprotective molecule in ischemic conditions, like stroke, for its antioxidant and anti-inflammatory actions. Several studies on animal models showed that melatonin therapy diminishes the toxicity induced by the glutamate-triggered increase in intracellular calcium in focal cerebral ischemia [37], increases the survival rate, and reduces neurodegeneration and post-ischemic neuronal loss [38]. The effectiveness of melatonin to improve neural viability not only following acute injury, but also as a long-term therapeutic agent, grants its position as a promising neuroprotector [39]. 

Given its high production in the neuronal mitochondria, melatonin is capable of interfering with neuronal dysfunction in the early stages of neurodegeneration [40]. Cellular senescence is accelerated by free radicals such as reactive oxygen (ROS) and nitrogen (RNS) species, which endorse caspase activation, cytochrome c release from mitochondria, and p53 protein cycle control [41,42] (Figure 1). Melatonin displays neuroprotection by diminishing cytochrome c release and caspase activation, and by reducing the activation of pro-inflammatory cytokines and thus, neurodegeneration [43]. Furthermore, melatonin lowers cell mortality rate and the mitochondrial dysfunction via the silent information regulator 1 (SIRT1) signaling pathway in cerebral ischemic lesions, in a similar way as it does in the myocardial injury [44].

Melatonin’s ability to prevent the neuronal death induced by kainate, a glutamate receptor agonist, supports its anti-excitotoxic activity [45]. Excitotoxicity is one of the most important pathophysiological mechanisms in ischemia/reperfusion (I/R) brain injury. The reactions following brain I/R injury are interlinked and often consist of calcium homeostasis alteration, an accumulation of reactive free radicals and activation of pro-inflammatory agents [46]. With regard to the in vitro experimental designs, melatonin was proven to counteract the destructive effects of hypoxia followed by reperfusion in primary cultures of rat cortical neurons [47]. 

An overview of melatonin’s mechanisms of action and protective effects is presented in Table 1.

As an endogenous and therapeutic molecule, melatonin ubiquitously exerts important benefits for human health. The present review summarizes significant and new data regarding the roles played by melatonin in homeostasis, development, and in oxidative-stress related pathologies, which favor allostatic overload. We chose to include some of the highest impact subjects on the matter, namely, the effects of melatonin on maternal-fetal health, cardiovascular diseases, neuroinflammation-related pathologies like degenerative neurological disorders, and respiratory distress caused by SARS-CoV-2 infection.

## 2. Melatonin Involvement in Maternal and Fetal Health

Melatonin’s role in pregnancy has been studied extensively lately and growing understanding became available of its physiological functions and its potential therapeutic use to improve maternal and neonatal outcomes. Melatonin is now considered a key signaling molecule between the mother and the fetus and a new potential candidate in the prevention of such complications as maternal preeclampsia or neonatal encephalopathy [73].

Melatonin exogenous supplementation during pregnancy could be beneficial both for the mother and the fetus, mainly but not only due to the antioxidant proprieties of melatonin [74].

### 2.1. Melatonin in Maternal-Fetal Signaling

During pregnancy, all maternal organs and systems sustain important changes to support the proper development of the fetus, in a continuous interplay between the maternal and fetal systems that promotes normal development. 

The pineal gland also undergoes structural and functional changes. Serum levels of melatonin increase in the second and third trimester, pick at term, and decline to normal immediately postpartum [75]. The specific cyclic night-time pattern of production for melatonin is maintained during normal pregnancy and lactation [30]. The placenta contributes to melatonin production but in a non-rhythmic manner [76]. Placental melatonin predominantly acts in an autocrine and paracrine fashion [50]. Because of its lipophilic structure, melatonin is transferred easily across the placenta into the fetal circulation [77]. The fetus relies on melatonin transferred from the mother since its pineal gland, though present in late gestation, begins functioning only after 3–5 months postnatally [78]. Fetal tissues present melatonin receptors, hence it is expected that maternal melatonin is involved in fetal growth and development [79]. In particular, the fetal brain displays melatonin receptors in multiple areas, thus maternal melatonin may play a role in the early stages of fetal neurodevelopment [80]. 

Recent evidence suggests that the circadian rhythm of the fetus and the newborn relay on the programming imprinted by the maternal circadian rhythms. Maternal melatonin is considered a key molecule for maternal-to-fetal signaling of circadian rhythm as it readily crosses the placenta and the levels in the maternal blood are mirrored in the fetal circulation, the mother seems to be the sole source and the fetus presents receptors in relevant areas. The circadian-generating systems in the fetus, particularly the suprachiasmatic nucleus, are considered to be under the influence of maternally derived melatonin [76]. Disturbances in the fetal circadian system may trigger long-term consequences. Interference to normal light/dark cycle in late pregnancy or during the perinatal period may have detrimental effects on the behavior and metabolic functions of the offspring, possibly contributing to conditions such as metabolic syndrome, obesity, attention deficit-hyperactivity disorder, or autism spectrum disorders [81,82,83,84].

Melatonin is excreted in the human milk early post-partum, especially at night, to compensate for the neonate lack of melatonin, as melatonin sustained rhythmic production starts only after several months or even later in preterm babies [85,86].

### 2.2. Melatonin for the Prevention of Maternal Pregnancy Complications

Alterations in normal melatonin production have been studied as a possible contributor to adverse pregnancy outcomes. A recent systematic review and meta-analysis by Cai at al. showed that pregnant women working rotating and night shifts, when compared to those working day-time only, have an increased risk of preterm birth (PTB), delivery of a small-for-gestational age baby, developing preeclampsia (PE) or gestational hypertension [87]. A possible explanation for these findings could be that reduced secretion of melatonin as a consequence of repeated disruption of the circadian rhythm and exposure to light in night workers could interfere with maternal and fetal hormone homeostasis, placental implantation, and fetal growth [55,88]. A recent study on mice where a model of induced maternal inflammation was used to mimic the clinical setting of preterm birth, showed that in mothers pre-treated with melatonin, there were fewer preterm deliveries and fewer cases of perinatal brain injuries in the pups [89]. The authors proposed that, because melatonin is considered generally safe in pregnancy, a study testing its effect for the prevention of PTB in humans should be initiated. Preterm birth (delivery of the baby before 37 weeks of gestation) is the big unresolved problem of modern obstetrics. It complicates around 7% of all pregnancies in industrialized countries and its incidence has remained the same in the last 30 years, even though obstetrical care has improved considerably [56]. While the outcomes of babies born preterm are improving due to the scientific and technical advancements in the field of neonatology, preventive, and therapeutic strategies for the mothers should be continually sought. The anti-inflammatory and antioxidant proprieties of melatonin are well recognized, and evidence from fundamental research studies is encouraging enough to consider testing melatonin in a randomized controlled trial (RCT) in the prevention of PTB in humans. This kind/type of trial would have to include around 1400 singleton pregnancies treated with either melatonin or placebo for a hypothetical reduction with 50% in the incidence of PTB (from 7% to 3.5%), with a power of 0.8 and alpha of 0.05. Other issues would regard the timing and duration of melatonin treatment (prenatal administration, during the first trimester or later in pregnancy), dosage, targeted population (low versus high-risk groups), and the route of administration. Melatonin supplementation is considered generally safe in humans. When given at a high dose by intravenous route, it does not induce somnolence or sedation or any other adverse events. Even with long-term administration, there are no associated adverse effects reported [54,57]. From studies with pregnant animals exposed to various doses of melatonin, there are no reported treatment-related side effects [90]. Even though data from human studies are limited, melatonin seems to have a good safety profile in human pregnancy, with no reported teratogenic effects [91].

Melatonin levels are altered in women with abnormally functioning placentas in preeclampsia and fetal growth restriction (FGR). Preeclampsia is characterized by new-onset hypertension after 20 weeks of gestation in association with maternal end-organ dysfunction and is a condition specific to human pregnancy with the only cure being the delivery of the fetus with the placenta. It is one of the leading causes of maternal mortality and morbidity, fetal death, and an important contributor to neonatal morbidity because of its need for iatrogenic preterm delivery. The pathogenesis of preeclampsia is likely to involve the mother, fetus, and placenta. In PE, there is defective placentation from early pregnancy, reduced blood perfusion in the placenta, hypoxia, and high levels of oxidative stress, with the release of trophoblast-derived factors (e.g., SFlt-1, soluble endoglin), which enter the maternal circulation and cause generalized endothelial dysfunction and an exaggerated inflammatory response [92]. For the fetus, this leads to growth restriction, preventing development to its full genetic potential. Serum levels of melatonin and placental distribution of its receptors are significantly reduced in pregnant women with preeclampsia or growth-restricted fetuses [30,93]. As a potent antioxidant and free-radical scavenger safe to be used during pregnancy, melatonin is a candidate drug for the prevention and treatment of PE and FGR. In a xanthine/xanthine oxidase (X/XO) placental explant model, melatonin was shown to reduce oxidative stress and enhance antioxidant markers, but it did not affect explant production of antiangiogenic factors (e.g., sFlt, soluble endoglin) [94]. In a model using primary trophoblast cell cultures, melatonin increased the expression of antioxidant enzymes and reduced the production of sFlt-1 [95]. In a clinical trial, 20 pregnant women diagnosed with early-PE (at the beginning of the third trimester) received 10 mg of melatonin three times daily from a median gestational age of 28 weeks until when the delivery was required for fetal or maternal indications. Compared to matched historical controls, those treated with melatonin, had a prolongation of pregnancy of ~6 days and a reduction of doses of antihypertensive medication [96]. While these effects are encouraging, more extensive, well-designed, randomized, double-blind, placebo-controlled trials are needed to test melatonin for the treatment of PE. Another approach to reducing the incidence of PE, would be to select a group of high-risk women who could possibly benefit from melatonin supplementation from the first trimester. This approach has been recently tested in a large RCT for which aspirin supplementation from 12 to 36 weeks of pregnancy in those high-risk, significantly reduced the incidence of PE [97].

FGR is frequently associated with PE. Chronic placental dysfunction, as seen in the placentas of growth-restricted fetuses, leads to intrauterine hypoxia, increases oxidative stress and ROS generation [98]. In a pilot phase I clinical trial in human pregnancy by Miller et al., melatonin (4 mg twice daily in a prolonged-release form) was administered to six pregnant women with severe early-onset FGR from a median gestational age of 27 weeks. Compared to historical control matched for diagnosis and gestational age placentas, oxidative stress was significantly reduced for those treated with melatonin [99].

### 2.3. Antioxidative Effects of Melatonin in Embryo-Fetal Development: Function and Therapeutic Approaches

Melatonin synthesis was identified at different stages of embryonic development and the main synthesis site has been reported to be the mitochondria [100] (Figure 1). Studies report that the melatonin level in the mitochondria was ~100-fold higher than the plasmatic concentration [48]. 

Melatonin plays a key role in embryonic development [101]. It has been shown that melatonin promotes embryo development in different species such as sheep, mouse, bovine, and pig [102,103,104]. All these positive effects exerted by the hormone on development have been attributed to its abilities to improve mitochondrial function and to reduce mitochondrial oxidative stress [33] combined with its function in the regulation of genomic methylation levels [105], which are key processes involved in embryonic development [106]. 

Melatonin is considered to act directly at the mitochondrial level, where it reduces free radical formation and also protects ATP synthesis, by stimulating key enzymatic complexes I and IV [50]. Direct scavenging of free oxygen and nitrogen species is also present at the mitochondrial level, by this protecting the membrane against disruption and supporting the continuity of the electron chain. Melatonin seems to protect mitochondrial DNA [49,107,108], and also prevents the opening of mitochondrial permeability transition pore (mPTP) in pathological conditions like traumatic brain injury and hypoxic ischemia. The opening of this pore allows macromolecules smaller than 1.5 kDa bypass the membrane, thus leading to mitochondrial swelling and death [109].

Melatonin’s role in the embryo and fetus is highly concentrated in nervous system development. The presence of a high number of melatonin receptors has been found in both nervous system organs and endocrine glands, thus supporting its function as a regulatory molecule. Melatonin receptors have been identified in human embryos and fetuses in the leptomeninges, cerebellum, thalamus, hypothalamus, brain stem, arcuate, ventromedial and mamillary nuclei. At brain stem level, there are melatonin receptor-positive areas in the nuclei of cranial nerves, such as oculomotor, trochlear, trigeminal motor and sensory, facial, and cochlear [110]. As melatonin’s main functions are represented by its antioxidative and DNA protective effects, one might assume that its highly expressed receptors in the nervous system organs during neurodevelopment point toward the same key-functions in the brain, during a period of time dominated by differentiation and subsequently high ROS production.

Hypoxic ischemia is a cause of severe brain injury in newborns and it represents one of the main causes of death in this age group and also a disability inducer [111]. During a hypoxic-ischemic injury, ROS production is increased massively, as that free species cannot be eliminated by antioxidant enzymes, leading to ROS accumulation. ROS accumulation is associated with structural changes to proteins, lipids, and DNA, thus leading to mitochondrial dysfunction, one of the main causes involved in the pathophysiology of this condition [112]. Mitochondria regulate the oxidative reactions standing at the basis of membrane potential maintenance and cellular ionic balance. Therefore, mitochondria play a crucial role in neonatal neurodegeneration. Up to date, the only efficient treatment is controlled hypothermia, but due to difficult clinical implementation and complications, it is not always applicable. New therapeutic strategies need to be searched, conceptualized, and methodically explored. 

Mitochondria-based therapeutic strategies have been reported in animal models, but have not been extensively tested in humans. Part of these treatment tactics originate from the roles that melatonin plays in mitochondrial physiology and pathophysiology. Along with its antioxidant properties, it has been shown to assert anti-inflammatory functions, by inhibiting the production of various anti-inflammatory molecules, like 8-isoprostanes, an important mediator involved in hypoxic-ischemic inflammation [52]. Melatonin also exhibits anti-apoptosis properties, by inhibiting Cytochrome C (Cyt C) release, Bax and Bad pro-apoptosis molecules production and preventing DNA disruption [53,113,114].

In animal studies, melatonin was shown to enhance the antioxidant enzyme glutathione peroxidase, and to reduce oxidative stress-induced DNA and lipids damage in fetal brain homogenates, when administered intraperitoneally in pregnant rats prior to 20 min bilateral utero-ovarian artery occlusion [40]. In another study, the prophylactic administration of melatonin to pregnant rats immediately prior to an acute ischemic episode and regularly throughout pregnancy, decreased ischemia-reperfusion-induced oxidative impairment in the premature fetal rat brain [41]. In addition, melatonin supplementation was shown to reduce maternal hyperthermia-induced embryo death, prevent preterm labor, and increase offspring survival in a mouse model of lipopolysaccharide (LPS)-induced inflammation [42,43].

Melatonin depletion through continuous light exposure during pregnancy was linked to intrauterine growth retardation in a rat model, which is avertible by maternal melatonin administration [44]. 

Melatonin toxicity has been assessed in human studies and is extremely low when including available data from children with severe neurological conditions or neonates with sepsis. One pilot RCT tested the clinical outcomes of melatonin in neonates with hypoxic-ischemic encephalopathy (HIE) [115]. The study included 45 newborns: 30 with HIE and 15 healthy controls. HIE infants were either treated with controlled hypothermia, or hypothermia plus melatonin. Serum nitric oxide (NO), plasma superoxide dismutase (SOD), and melatonin levels were measured for the 45 neonates on admission and at 5 days since the beginning of the study. The study showed that the HIE groups had increased melatonin, SOD, and NO. After 5 days, the melatonin/hypothermia group had a significantly higher level of melatonin and a decrease in NO and SOD. This group also had a lower rate of seizures on follow-up electroencephalogram and less extensive white matter abnormalities on MRI. Importantly, at 6 months, the melatonin/hypothermia group had a significantly better survival rate. Melatonin appears to be safe and beneficial in HIE treatment. However, larger randomized controlled trials are required before melatonin can be approved for usage as a neuroprotective molecule.

## 3. Melatonin’s Role in Programming Adult Cardiovascular Homeostasis and Allostasis

Melatonin accompanies the development and homeostasis of the cardiovascular system all throughout life with distinctive effects in both the perinatal and adult periods. As previously discussed, the most prominent protective mechanism lies in its antioxidant potential [19,116]. In the field of cardiovascular effects, melatonin protects against ischemia/reperfusion injury (IRI) [62,117,118,119,120,121] and hypertension [122,123,124], as an endogenous effect, nutritional supplement, or acute parenteral administration [125,126]. In addition, it appears that other promising effects such as anti-apoptotic, anti-inflammatory [127], preconditioning [118,128], myocardial infarction [129,130,131], and even an implication in heart failure [132,133,134,135] have been documented. However, it seems than in some conditions, melatonin can have deleterious effects, as exaggerated collagen and glycosaminoglycans deposition and increased size in post-myocardial infarction scar have also been reported [136,137]. A reduction in cardiovascular risk factors such as diabetes and hyperlipidemia could be of use in preventive cardiovascular care. Even before disease manifests, melatonin contributes to allostasis, as it is involved in regulating prediabetes, inflammation, and lipid metabolism [124]. These risk factors precede conditions like atherosclerosis, coronary artery disease, cerebrovascular disease, etc. Slowing-down of these processes through the positive effects of melatonin could be an effective prophylactic therapy. The allostatic effects are not efficient in some individuals, as melatonin levels decrease with age and with cumulating risk factors [124]. If there is a threshold below which these positive effects are no longer observed, or if there is a specific moment in the day when the melatonin level should surpass that threshold, it is still not clear.

### 3.1. Prenatal and Neonatal Cardiovascular Effects

Melatonin has been implicated in prenatal programming in more than one aspect. The antioxidant role is significant for babies at risk of birth asphyxia, as the new-born myocardium has reduced antioxidant defense [138]. In addition, in an experimental setting of intrauterine growth restriction, melatonin counteracts the vascular and myocardial impairment seen in the untreated group [139].

Melatonin was shown to prevent the development of hypertension by increasing the level of nitric oxide (NO) [140], although the exact mechanism has not yet been agreed upon. Supposedly, the antioxidant properties of melatonin might aid in restoring the NO/ROS balance against programmed hypertension. The offspring subjected to high methyl-donor diet-induced programmed hypertension also displayed a lower SDMA (symmetric dimethylarginine) plasma level, which is an endogenous NO inhibitor, following maternal melatonin administration [141,142]. In a rat model, chronic photoperiod shifts (CPS) during 85% of the pregnancy were shown to generate increased systolic BP at night and higher heart rate variability at rest in the young adult offspring (raised and tested under normal light/dark conditions) [143].

### 3.2. Antioxidant and Anti-Inflammatory Roles in Cardiovascular Pathologies

The roles of antioxidant and anti-inflammatory agent are intertwined in the case of melatonin. The anti-inflammatory role of melatonin in clinical practice is still mechanistically challenged. Melatonin reduced free radical production, lipid peroxidation, and IL-6 levels caused by bacterial lipopolysaccharide in vitro [116]. In a human experimental sepsis model, melatonin reduces IL-1β, but does not affect IL-6 and TNF-α [144]. However, obese women who received melatonin supplementation showed lower TNF-α and IL-6 levels, a fact that was not demonstrated in the previously mentioned study [144]. The differences between the two studies were that in the sepsis model study, melatonin was given in a single, higher dose of 100 mg, and in the obesity trial, the subjects received daily doses of 6 mg over 40 days (the overall dose was 2.4× higher). Another clinical study mentions higher IL-6 levels, and lower melatonin concentration in myocardial infarction patients compared to the control group [145]. The authors suggest that there is a causal relationship between melatonin and IL-6 levels. Different research stresses that the C-reactive protein (CRP) is increased in ST-elevation myocardial infarction (STEMI) patients compared to healthy subjects. This correlates with a diminished surge in the night-time melatonin levels and also with an increased risk of adverse events in the following six months [146]. The link between CRP and melatonin is represented by IL-6. This interleukin induces CRP production and also shows an increase in relation to melatonin [145]. Inflammation plays an important role in the pathogenesis of the atherosclerotic disease [147,148]. Thus, the anti-inflammatory properties of melatonin should be exploited in a preventative capacity.

### 3.3. Effect on Adult Cardiovascular Risk Factors

**Figure 3:**
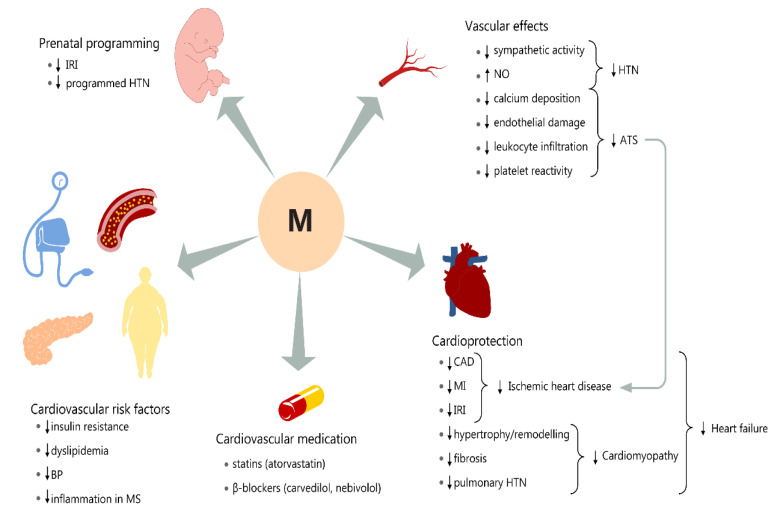
Cardiovascular effects of melatonin. ATS: atherosclerotic disease, BP: blood pressure, CAD: coronary artery disease, IRI: ischemia/reperfusion injury, HTN: hypertension, MI: myocardial infarction, MS: metabolic syndrome, NO: nitric oxide.

The *metabolic syndrome* is a pool of cardiovascular risk factors, like obesity, hypertension, dyslipidemia, and pre-diabetes. It is also accompanied by low-grade adipose tissue inflammation, which acts as a precursor to insulin resistance and diabetes. Patients with metabolic syndrome have a 1.5–2.5 higher risk of cardiovascular death. Melatonin proved to be useful in managing the metabolic syndrome components mainly through its antioxidant, anti-inflammatory, and immunomodulatory actions (Figure 3). Along with their main use in sleep disorders, melatonin’s analogs have been shown to be beneficial in managing metabolic syndrome components in rodents. Thus, ramelteon lowered blood pressure values and body weight [149], while piromelatine, a newer melatonin analog, improved insulin resistance and stabilized the metabolic profile [150].

*Age* is also a cardiovascular risk factor and unfortunately, melatonin production progressively decreases with age, in the pineal gland, as well as in the other extra-pineal tissues (heart, liver, spleen). Consequently, cardiovascular protective mechanisms diminish. Inflammation is a key process in aging and in age-related diseases, thus, the term *inflammaging* was introduced to highlight this strong connection [124]. Melatonin levels were inversely correlated with inflammation in a study on more than 1000 elderly subjects [151], while experimental data suggest that melatonin is also involved in slowing of myocardial aging [152,153].

In elderly individuals, a connection between night-time decreased melatonin secretion, and *hypertension* has been established [154]. This relationship is not maintained in patients already treated for hypertension. In addition, melatonin reduces nocturnal blood pressure values [155]. Studies in sleep patterns showed susceptibility to hypertension in patients with longstanding insomnia [156].

Nitric oxide (NO) production causes vasodilation and reduction of blood pressure values. Melatonin associated NO increase has been documented in certain circumstances [122,123,157]. However, other studies report the inhibition of NO production, as part of the antioxidant role of melatonin.

Oxidative stress is a major factor in the development of *diabetic* complications. A potent antioxidant as melatonin has proven beneficial effects on ROS decrease and beta-cell protection in diabetic patients [158]. It also contributes to a decrease in insulin resistance [159] and melatonin supplementation has shown positive results in some clinical studies [158].

Melatonin improves *dyslipidemia* and *body weight* in several animal models of hyperadiposity [124]. The reduction of oxidized low-density lipoproteins and triglycerides was also linked to melatonin administration [160]. As previously mentioned, melatonin demonstrates both an anti-inflammatory and antioxidative effect in obesity [161,162]. Part of these properties are attributed to activation of the silent information regulator 1 (SIRT1) [162]. Furthermore, obese women with low melatonin levels have a higher risk of myocardial infarction [163]. Moreover, melatonin is abundant in the gastrointestinal system, approximately 400 times the amount found in the pineal gland [164]. Melatonin reprograms the gut microbiota and improves lipid metabolism [165].

### 3.4. Vascular and Myocardial Effects

Atherosclerosis (ATS) is the consequence of cholesterol build up and chronic inflammation, in the context of a dysfunctional endothelium. Endothelial dysfunction is partially caused by insufficient NO production via eNOS synthesis, and partially by free radicals such as ROS, significantly involved in vascular disease pathogenesis. Thus, the association between a hypocholesterolemic agent, such as statins and melatonin seem logical. Indeed, a study using both substances proved that melatonin reduces the oxidative stress and improves the statins’ capacity to stimulate eNOS and increase NO production with consequent vasodilation [116].

Melatonin decreases the sympathetic activity and shifts the neurovegetative balance in favor of the parasympathetic system [122]. This is reinforced by the fact that plasma melatonin levels rise as a counter-regulatory mechanism in hypertension triggered by sympathetic overstimulation [123].

Melatonin is also vasoprotective by dampening the inflammation subsequent to the atherosclerotic process (Figure 3) [166]. Through interference with ATS progression, melatonin also possibly contributes to slower development of ischemic heart disease [127]. Moreover, it inhibits calcium accumulation, leucocyte infiltration, and endothelial damage [167,168]. In addition, it has antithrombotic effects through a reduction in platelet reactivity [129].

The cardiac insult produced by experimental myocardial infarction induces an increase in pineal melatonin secretion [169]. This suggests that endogenous melatonin displays a cardioprotective effect (Figure 3). On the other hand, it seems that in patients with coronary artery disease, this mechanism is impaired.

The day/night pattern of melatonin secretion and the inverse correlation to cortisol levels have a connection to the timing of certain adverse events. Myocardial infarction, sudden cardiac death, and even malignant arrhythmias occur early in the morning when melatonin levels are low [170,171]. This relationship could be confounded with the diurnal pattern of sympathetic system activation, which is also influenced by melatonin. It was demonstrated that corticosterone has a dual effect on melatonin production, influenced by the sympathetic system. This effect is based on the stimulation pattern of adrenoceptors, only beta_1_, or beta_1_ and alpha_1_. Thus, sympathetic system activation is necessary for the control of melatonin synthesis in certain conditions and vice-versa [123,172]. A recent study shows norepinephrine (NE) levels accompanying a myocardial infarction are indeed influenced by melatonin administration [173].

Melatonin has demonstrated its potential role in protecting the myocardium against *ischemia/reperfusion injury* in multiple settings [62,117,120,121,128]. At low concentrations (4 mg/kg/day), it activates several signaling pathways that offer protection against IRI [20,174]. These pathways include JAK-STAT3, and nuclear factor erythroid 2-related factor 2 (Nrf2) (Table 1).

For the coronary artery bypass graft (CABG) patients, a five-day melatonin administration (10 or 20 mg/day) before surgery showed beneficial effects in a dose-dependent manner, as compared to placebo [65,136], recommending melatonin as a preventive method.

Some studies showed that melatonin produces a decrease in the myocardial scar, reduced fibrosis [134], and remodeling after myocardial infarction [130]. In the MARIA (Melatonin Adjunct in the acute myocaRdial Infarction treated with Angioplasty) study on reperfused STEMI patients, melatonin reduces infarct size 40% if administered in the first 2.5 h after symptom onset [125,129]. In addition, melatonin improves reverse remodeling after cardiac resynchronization therapy [132]. Melatonin has lower levels not only in patients with myocardial infarction, but also in dilated cardiomyopathy, and is correlated with cardiac output [133]. 

Cardiotoxicity is a known side effect of cytostatic therapy. Melatonin protects against doxorubicin-induced cardiotoxicity by decreasing apoptosis and oxidative stress [175]. 

Insomnia is a common side effect of first and second-generation beta-blockers, a common cardiovascular medication. Newer molecules have been tailored as not to modify the melatonin levels, i.e., nebivolol and carvedilol vs. the other beta-blockers [176,177].

### 3.5. Neutral and Deleterious Effects

After several promising small animal studies on the effect of melatonin on MI size, a large animal model failed to prove melatonin’s role in MI cardioprotection, when administered prior to myocardial reperfusion either intravenously or intracoronary, thus raising questions on the relevance of melatonin in this context [126]. Whether the lack of efficacy is due to an inappropriate dose, timing or administration route is yet to be investigated. This initial translational fiasco should point to scrupulously testing and tweaking any novel therapy before going further.

Patients with longer-lasting ischemia symptoms (>3.5 h) exhibited an increase in MI size after melatonin administration compared to the placebo group [131]. This effect, as well as lower ejection fraction and higher left ventricle end-diastolic and systolic volumes [125], might be explained by reports on collagen and glycosaminoglycan deposition following melatonin treatment [178,179,180].

Despite numerous clinical and preclinical trials, authors with vast experience in the field of ischemia/reperfusion injury, preconditioning, and cardioprotection do not believe melatonin to be relevant to MI scar reduction in STEMI reperfused patients [137].

## 4. Melatonin Effects in Neuronal Inflammation and Degenerative Neurological Diseases

Neurological diseases represent the second most common cause of death with a mortality rate of 16.8% worldwide, according to the Global Burden of Disease Study 2015. In the last 25 years, the burden of neurological disorders has increased worldwide. Alzheimer’s disease (AD) and other dementias are one of the four major contributors and account for up to 10% of the disability-adjusted life-years (DALYs) [181]. 

It was shown that generation or aggravation of various neuropathological conditions are accompanied by a low-grade inflammation and blood–brain barrier impairment, and these conditions could be associated with alteration of sleep and melatonin secretion [182].

The low-grade inflammatory state in the aged brain, known as *inflammaging,* mainly refers to an excessive production of pro-inflammatory cytokines and proteins such as integrin alpha-M (CD11b), IL-1β, IL-16, and TNF-α and downregulation of agents such as brain-derived neurotrophic factor (BDNF), which were significantly altered in aged-animal models [69]. In lipopolysaccharide (LPS)-treated animals, neuroinflammation is augmented by enhanced oxidative stress, pro-inflammatory cytokines, such as TNF-α, IL-6, IL-1β, and NF-κB phosphorylation.

Various studies showed that exogenous melatonin administration reduces the damaging effects of LPS on nerve cells [183,184]. In a recent review, the close relationship between neuroinflammation and neurodegeneration has been linked by the dysregulation of glial-neuronal communications. Thus, altered signaling promotes glial activation, NF-κB activation and release of proinflammatory cytokines that further determine overactivation of protein kinases and favor amyloid beta and tau proteins accumulation [185]. Together with the aging process, the risk for neurodegenerative diseases such as mild mild cognitive impairment (MCI) and Alzheimer’s disease increases.

Multiple pathological patterns that lead to neurodegeneration have been described, the most popular ones being oxidative stress, mitochondrial dysfunction, low degree of inflammation, excitotoxicity, and impaired pruning mechanisms that lead to accumulation of neurotoxic aggregates such as beta amyloid oligomers [186]. Given the melatonin’s main characteristics, as a circadian rhythm regulator and as a potent antioxidant, multiple studies have investigated its potential role in preventing neurodegeneration. Melatonin secretion is regulated by the suprachiasmatic nucleus, according to environmental light/dark cycles. Its main role is to synchronize the phase and the amplitude of the peripheral biological rhythms. On the other hand, melatonin’s cytoprotective properties allow it to partially reverse the inflammatory damage seen in neurodegenerative disorders and aging [187].

However, notwithstanding the antioxidant and anti-inflammatory characteristics of melatonin, some studies have brought significant data regarding its efficacy against brain injury in humans. Zhao et al. recently demonstrated that melatonin might have a protective effect on cerebral I/R injury in both rats and humans on cerebral I/R-related injury after carotid endarterectomy surgery [39].

Melatonin has been shown to be beneficial in cerebral ischemia. In addition, its antioxidant properties might help slow down neuronal aging, especially when considering its low blood levels in the elderly. In experimental models of Alzheimer’s and Parkinson’s diseases, the neurodegeneration observed is partially prevented by melatonin [188]. Generally, the progression of these neurological conditions and other forms of dementia is associated with low melatonin secretion [189]. 

### 4.1. Alzheimer’s Disease

Alzheimer’s disease is the most common neurodegenerative disease of the brain. The pathogenesis of AD is characterized by the presence within the nerve cell of fiber-like deposits of the microtubular tau protein and by the accumulation of amyloid beta. The antioxidant role of melatonin and inhibiting action on oxidative stress was acknowledged in various neurodegenerative disorders such as Alzheimer’s or Parkinson’s disease. In AD, the intracellular accumulation of abnormal proteins become senile plaques consisting of tau filaments and amyloid beta proteins which disrupt the neuronal homeostasis and damage axonal conduction [190]. Oxidative stress causes structural changes in cellular DNA, proteins, and lipids that eventually form damaging protein deposits. Physiologically, melatonin is released directly into the cerebrospinal fluid, thus having a higher concentration in comparison with its blood level. In patients with AD, melatonin concentration in the cerebrospinal fluid is decreased, suggesting that a deficiency of this molecule is correlated with the pathophysiology of this condition [191]. During sleep, the elimination of amyloid beta molecules increases considerably. The disruption of the circadian rhythm in AD, hence, might favor the progression of the disease by slowing the amyloid beta clearance. Moreover, melatonin effectively inhibits amyloid beta synthesis and tau filament formation [192].

Mild cognitive impairment and AD are frequently associated with low melatonin circulating concentrations in animal models [193]. With regard to clinical trials involving melatonin administration to humans, recent studies support the efficacy of melatonin in improving cognitive deterioration and reducing the so-called “sundown” effect [194]. The sundown phenomenon implies the aggravation of specific symptoms such as disorganized thinking, agitation, emotional lability, and attention deficit in the evening.

### 4.2. Parkinson’s Disease

Parkinson’s disease (PD) comprises the progressive neurodegeneration of dopamine containing neurons located in the pars compacta of the substantia nigra. The pathophysiology of PD is multidimensional, with no existing treatment meant to stop the development of the disease. Current medication is symptomatic and designed to alleviate the motor component of the disease. However, PD affects multiple neurotransmitter systems and involves a non-motor component regarding the autonomic nervous system. Most common symptoms include genitourinary impairment, decreased bowel movements, respiratory and cardiovascular disorders, neuropsychiatric and sleep-related disorders [97]. The pathological patterns which cause the neurodegeneration process in PD are represented by neuro-inflammation, microglial activation, and lymphocytic infiltration. By adjusting the activity of antioxidative enzymes like SOD, mitochondrial complex-I activity, and glutathione (GSH), melatonin decreased oxidative stress in a rat model of PD disease [195]. A study reported that administration of melatonin improved non-motor disorders in patients with PD [196].

The mechanisms through which melatonin helps averting neurodegeneration in Parkinson’s are closely related to its anti-excitotoxic activity and scavenger properties. Melatonin was efficient in inhibiting α-synuclein assembly and in softening kainic acid-induced neurotoxicity [197] and arsenite-induced apoptosis [198]. Moreover, it was proved to block α-synuclein fibril formation and to disrupt pre-synthesized fibrils by hindering protofibril creation and protein secondary structure transitions, while reducing α-synuclein cytotoxicity [198,199].

## 5. Melatonin as an Adjuvant in Respiratory Distress Caused by SARS-CoV-2

In light of the recent global health events, the search for anti-inflammatory molecules has brought attention to melatonin for its combined anti-inflammatory and antioxidative effects for the treatment of SARS-CoV-2 respiratory infection. As a protector against acute respiratory distress syndrome (ARDS), melatonin proved to be a good adjuvant in respiratory diseases, while also improving the cardiac function in pulmonary arterial hypertension [14]. In a recent report on ramelteon, a melatonin receptor agonist, it was demonstrated that melatonin averts volume induced lung injury in a rat model, by decreasing lung edema, nuclear factor kappa light chain enhancer of activated B cells (NF-κB) activation, iNOS levels, apoptosis, and IL-10 production [71]. Moreover, melatonin prevents lung fibrosis through multiple mechanisms. It interferes with the fibrogenic response, activation of effector cells, elaboration of the extracellular matrix, and its active deposition. Melatonin also diminishes bleomycin-induced lung fibrosis in mice by inhibiting transforming growth factor β 1 (TGF-β1) [200,201] and it is protective in radiation-induced lung injury by suppressing the NOD-like receptor protein 3 (NLRP3) inflammasome [70,202]. Through its antioxidant and anti-inflammatory effects, melatonin is beneficial in pulmonary hypertension [203,204,205], enhances the NO-independent acetylcholine’s vasodilatory effect, decreases the systolic pressure of the right ventricle, and prevents right ventricular hypertrophy [206]. By diminishing TGF-β1 activity, apoptosis, and endoplasmic reticulum stress via Sirtuin-1 upregulation in animal studies, melatonin could be protective in chronic obstructive pulmonary disease (COPD) [72,207]. It also decreases dyspnea and 8-isoprostane in the exhaled air, in humans [208].

COVID 19 is a disease induced by the human body infection with the SARS-CoV-2 virus, which affects especially the lungs, but also the gastrointestinal and nervous system. Respiratory failure in this type of infection is associated with severe hypoxemia. Clinically, two phenotypes were described: L-type and H-type. The L-type is characterized by low elastance (high compliance), low ventilation-to-perfusion ratio (V/Q), low lung weight, low recruitability, whereas the H-type is accompanied by high elastance, high right-to-left shunt, high lung weight, and high lung recruitability [209]. Another important feature of this disease is the presence of venous thrombosis and lung micro-thrombosis [210].

The ARDS present in COVID-19 is induced by the “cytokine storm”. Dendritic and epithelial cells infected with SARS-CoV-2 produce several inflammatory cytokines (IL-1β, Il-2, Il-6, Il-8, TNF-α) and chemoattractants (monocyte chemoattractant protein-1 (MCP-1)/CCL-2 (C-C Motif Chemokine Ligand 2), CCL-3, CCL-5, interferon γ (IFN_γ_)–induce protein 10 (IP-10)) that attract other neutrophils and monocytes/macrophages resulting in an excessive inflammatory response. These processes lead to alveolar-capillary membrane damage in the lungs [211]. Other mechanisms reported in this type of respiratory failure are vascular leakage, apoptosis of epithelial and endothelial cells.

An intuitive reason for melatonin’s therapeutic effect in COVID-19 is based on its pleiotropic traits (anti-inflammatory, antioxidant, and immunomodulatory) and the observation that children and young patients, known to have higher melatonin levels, show milder forms of disease than the elderly [212]. In a review on the effects of melatonin in COVID 19, Zhang et al. describe its multiple theoretical benefits. Its anti-inflammatory effects are mediated by the proper regulation of Sirtuin-1, suppression of NF-kB, and a reduction of pro-inflammatory cytokines [213]. The antioxidant effect is performed by down-regulating NOS and direct scavenger. The immunomodulatory effect is mediated by the regulation of lymphocytes, macrophages–antigen-presenting cell (APC) activity, and inhibition of NLPR-3 inflammasome.

Melatonin has a vasodilatory effect on the pulmonary circulation and decreases pulmonary blood pressure. However, this effect does not seem to improve the V/Q mismatch with systemic administration in L-type infection, because theoretically, it could increase the hypoxemia [204,205]. Intratracheal administration might be the best choice, to induce a NO-like effect and consequently improve the perfusion in ventilated alveoli. This type of melatonin administration is safe and has proven diminished inflammation via NLPR-3 inflammasome inhibition [214]. Nevertheless, there is a debate if NO administration is really beneficial due to the supposed pulmonary vasoplegia present in the L-type ARDS [209]. Moreover, a lack of pulmonary vessels’ response to NO makes the administration of melatonin probably ineffective.

Melatonin decreases D-dimers levels in stress-induced coagulopathy [215], and has a suppressive effect on burn-induced coagulopathy. However, melatonin was not able to prevent the disseminated intravascular coagulation induced by sepsis, and presumably its administration might not contribute to the prevention of COVID-19 related thrombosis [216].

Melatonin through its anti-inflammatory, antioxidant, and immunomodulatory effects is a perfect candidate as a treatment add-in in COVID-19 but its efficacy is yet to be demonstrated in clinical trials. One certain effect is that it reduces the ICU delirium and this could be a strong recommendation for long-term sedated patients with COVID-19 requiring mechanical ventilation [217].

## 6. Promising Clinical Targets And Pending Questions

Melatonin has been administered to humans by intranasal, transdermal, oral, transmucosal, and intravenous routes, however, it is not known whether these routes are suitable for pregnancy and if melatonin’s pharmacokinetics is different in pregnant women [57]. These are questions that would need to be addressed if we were to think of melatonin as a potential intervention to prevent pregnancy complications. Importantly, there is need for solid data not only on the safety and pharmacokinetic profile of exogenously administered melatonin during pregnancy but ideally also on the long-term follow-up for the babies exposed in utero.

The Mediterranean diet has been confirmed as protective for individuals at high risk of cardiovascular events [218]. Part of this beneficial effect might be attributed to the melatonin content of some of the components of this type of diet, such as red wine, pistachio nuts, olives, fish, and seasonal fruits [219,220]. Concerning exogenous melatonin administration, a relevant question is whether dietary melatonin is enough or a higher dose would be necessary for an efficient cardioprotection. Furthermore, the issues regarding the ideal administration route and timing are still unresolved.

Is there a specific endogenous level below which melatonin becomes inefficient as a protective mechanism and an exogenous supplementation might be beneficial? If so, what is that level, what are the efficient doses and when should they be implemented? All these questions are still open-ended.

Additionally, melatonin analogues (ramelteon, agomelatine, tasimelteon, piromelatine), with a long-lasting effect are currently being explored in clinical trials. Interestingly, the doses used for these analogues are significantly higher than for melatonin. Thus, maybe it is justified to also test higher doses of melatonin (50–100 mg/day) in order to assess its role in cardiovascular prevention [124].

Although very promising in preclinical trials, melatonin seems to fail to deliver clinically. Melatonin failed the translational trial in established cardiovascular disease [137], but nevertheless acts on several cardiovascular disease risk factors (diabetes, obesity, hypertension, dyslipidemia). Should this be a clue it ought to be included in the prevention guidelines for patients at risk of developing cardiovascular diseases?

As both the neurodegenerative disorders and cardiovascular diseases represent a potential target for melatonin’s beneficial prophylactic effects, maybe a joint guideline for patients at risk would be of clinical use.

Fortunately, clinical trials involving melatonin administration do not cease to appear. Even in higher doses, melatonin proved to have a satisfying safe profile and elicited a nontoxic effect. Hence, its applications as a neuronal protector were exploited both in acute disease, especially brain ischemia and in degenerative conditions, such as Alzheimer’s, Parkinson’s, or Huntington’s diseases. The pathological accumulation of intracellular proteins that later form senile plaques in AD is reduced by melatonin. Furthermore, its dual dimension, as a chrono-biological regulator and antioxidant was critical in improving cognitive deterioration and reducing the “sundown” effect in AD. Its ability to adjust antioxidative enzymes rendered a lowering of neuronal oxidative stress in PD in animal models, whilst yielding favorable results in human trials with respect to non-motor symptoms in PD.

Here and now, an important question is whether melatonin will prove itself useful in managing the cytokine storm associated to COVID 19. A better mechanistic understanding of the disease will either support the initiation of research on the matter or discourage it altogether. At the moment, the only clear recommendation for the use of melatonin is the association of 6–12 mg at night, according to the recently released MATH+ protocol (Methylprednisolone, Ascorbic acid, Thiamine, Heparine + melatonine, zinc, vitamin D, famotidine, magnesium) [221].

As more and more data accumulate around the subject of melatonin and its clinical use, it becomes abundantly clear that even if some useful properties are “lost in translation”, there are still a lot of questions that, when answered, will perhaps be a breakthrough.

## Figures and Tables

**Table 1:** Overview of the mechanisms of action and the protective effects of melatonin.

	Mechanism	Effect			Study Type
**Maternal & Fetal Health**	Improves mitochondrial function, reduces mitochondrial oxidative stress [48]	Promotes embryo development			In vivo
	TET genes function [36]	Regulation of genomic DNA methylation levels			In vitro
	Counteracts the effects of *Aanat* knockdown [33]	Protects fertilization and tissue differentiation			In vitro
	Direct scavenger of hydroxyl groups and nitrogen reactive species [49]	Detoxification of superoxide anions			In vitro
	Mitochondrial complexes I and IV [50]	Protects ATP synthesis			In vitro
	Prevents pathological opening of mPTP [51]	Protects mitochondrial DNA			In vitro
	Inhibits anti-inflammatory molecules, like 8-isoprostanes [52]	Reduces hypoxic-ischemic inflammation			In vivo
	Inhibits Cytochrome C release [53]	Anti-apoptotic			In vitro
	Increases Bax and Bad pro-apoptosis molecules production [54]	Anti-apoptotic Prevention of DNA disruption			In vitro
	Enhances glutathione peroxidase levels [55]	Antioxidant			In vitro
	Not known [56]	Increases offspring survival in a model of lipopolysaccharide-induced inflammation			In vivo
	Not known [57]	Prevents intrauterine growth retardation associated with continuous light exposure during pregnancy			In vivo
	Increases plasma NO and SOD levels [58]	Beneficial in hypoxic-ischemic encephalopathy of the newborn			In vivo
**Cardiovascular**		Immediate	Intermediate	Final	
	Inhibits MPTP opening [59,60]	Decrease apoptosis	Decrease cell death	Prevent/reduce myocardial IRI	In vitro
	Inhibit Cyt C release [61]	Decrease apoptosis	Decrease cell death		In vitro
	Activates JAK/STAT3 [62]	Decrease Bax, Increase Bcl	Decrease apoptosis		In vitro
	Improves TAC [60,61]	Scavenger activity Increase endogenous antioxidant capacity Stop cardiolipin peroxidation	Decrease oxidative damage		In vitro
	Improves calcium handling [63]	Ca^2+^-calmodulin modulation	Decrease cell death, reduce apoptosis		In vivo
	Nrf 2 [64,65]	Transactivate HO-1	Decrease inflammation and oxidation		In vivo
	iNOS inhibition [66,67]	Lower NO levels	Decrease oxidative stress		In vivo In vitro
	Inhibits inflammatory cytokine release [67]	Decrease of TNF-α, IL-1β, IL-6	Decrease inflammation		In vitro
	Stimulates anti-inflammatory cytokines [68]	Increase of IL-10	Decrease inflammation		In vivo
**Neuroinflammation**	Maintains the levels of parvalbumin and hippocalcin [37]	Prevents neuronal death in cerebral ischemia			In vivo In vitro
	Decreases NO, peroxynitrite formation [38]	Reduces hyperactivity linked to neurodegeneration induced by cerebral ischemia and reperfusion; Reduces PARS and brain edema			In vivo In vitro
	Activation of SIRT1 signaling [44]	Reduced infarct volume, lowered brain edema, increased neurological scores in IRI			In vivo In vitro
	Prevents accumulation of free radicals [47]	Counteracts the destructive effects of NMDA or hypoxia/reperfusion			In vitro
	Decreases pro-inflammatory cytokines IL-1β, IL-6, and TNF-α in PFC [69]	Attenuates neuroinflammation in the aged mouse brain			In vivo In vitro
**Respiratory**	Suppresses the NLRP3 inflammasome [70]	Protectects against radiation-induced lung injury			In vivo In vitro
	Decreasies lung edema and reduces NF-κB activation, enhances the secretion of IL-10 [71]	Averts volume induced lung injury			In vivo In vitro
	Sirtuin-1 upregulation [72]	Diminishes TGF-β1 activity, apoptosis, and endoplasmic reticulum stress			In vivo In vitro

AANAT: aralkylamine *N*-acetyltransferase; Cyt C: cytochrome C; DNA: deoxyribonucleic acid; IRI: ischemia reperfusion injury; JAK/STAT3: Janus kinase 2/signal transducers and activators of transcription 3; iNOS: inducible nitric oxide synthase; HO-1: heme oxygenase-1; IRI: ischemia-reperfusion injury; mPTP: mitochondrial permeability transition pore; NO: nitric oxide; NF-κB: nuclear factor kappa light chain enhancer of activated B cells NLRP3: NOD-like receptor protein 3 Nrf 2: nuclear factor erythroid 2-related factor 2; PARS: poly (ADP-Ribose) synthetase (PARS); SIRT1: Silent information regulator 1; SOD: superoxide dismutase; TAC: total antioxidant capacity; TET = ten-eleven-translocation; PFC: pre-frontal cortex.
